# Supplementary material for: COZOID: contact zone identifier for visual analysis of protein-protein interactions
Source: BMC Bioinformatics. 2018 Apr 6;19:125. doi: 10.1186/s12859-018-2113-6 (PMC5889581; doi:10.1186/s12859-018-2113-6)
Supplement: Supplementary file 2 — Software build. Executable binary file of the software tool. (ZIP 118,784 kb) [file 12859_2018_2113_MOESM2_ESM.zip › cozoid/java/docs/beansbinding-1.2.1-doc.zip!/index-files/index-10.html]

J-Index (Beans Binding)


---


|  |  |  |
| --- | --- | --- |
| **Overview** \| Package \| Class \| Use \| **Tree** \| **Deprecated** \| **Index** \| **Help** | |  |
| **PREV LETTER**   **NEXT LETTER** | **FRAMES**    **NO FRAMES**     **All Classes** |  |


A B C D E F G H I J K L M N O P Q R S T U V W 

---


## **J**

**JComboBoxAdapterProvider** - Class in org.jdesktop.swingbinding.adapters: **JComboBoxAdapterProvider()** - Constructor for class org.jdesktop.swingbinding.adapters.JComboBoxAdapterProvider: **JComboBoxAdapterProvider.Adapter** - Class in org.jdesktop.swingbinding.adapters: **JComboBoxBinding**<E,SS,TS> - Class in org.jdesktop.swingbinding: Binds a `List` of objects to act as the items of a `JComboBox`. **JComboBoxBinding(AutoBinding.UpdateStrategy, SS, Property<SS, List<E>>, TS, Property<TS, ? extends JComboBox>, String)** - Constructor for class org.jdesktop.swingbinding.JComboBoxBinding: Constructs an instance of `JComboBoxBinding`. **jj\_nt** - Variable in class org.jdesktop.el.impl.parser.ELParser: **jjFillToken()** - Method in class org.jdesktop.el.impl.parser.ELParserTokenManager: **jjnewLexState** - Static variable in class org.jdesktop.el.impl.parser.ELParserTokenManager: **jjstrLiteralImages** - Static variable in class org.jdesktop.el.impl.parser.ELParserTokenManager: **jjtAddChild(Node, int)** - Method in interface org.jdesktop.el.impl.parser.Node: This method tells the node to add its argument to the node's list of children. **jjtAddChild(Node, int)** - Method in class org.jdesktop.el.impl.parser.SimpleNode: **JJTAND** - Static variable in interface org.jdesktop.el.impl.parser.ELParserTreeConstants: **JJTBRACKETSUFFIX** - Static variable in interface org.jdesktop.el.impl.parser.ELParserTreeConstants: **JJTCHOICE** - Static variable in interface org.jdesktop.el.impl.parser.ELParserTreeConstants: **jjtClose()** - Method in interface org.jdesktop.el.impl.parser.Node: This method is called after all the child nodes have been added. **jjtClose()** - Method in class org.jdesktop.el.impl.parser.SimpleNode: **JJTCOMPOSITEEXPRESSION** - Static variable in interface org.jdesktop.el.impl.parser.ELParserTreeConstants: **JJTDEFERREDEXPRESSION** - Static variable in interface org.jdesktop.el.impl.parser.ELParserTreeConstants: **JJTDIV** - Static variable in interface org.jdesktop.el.impl.parser.ELParserTreeConstants: **JJTDOTSUFFIX** - Static variable in interface org.jdesktop.el.impl.parser.ELParserTreeConstants: **JJTDYNAMICEXPRESSION** - Static variable in interface org.jdesktop.el.impl.parser.ELParserTreeConstants: **JJTEMPTY** - Static variable in interface org.jdesktop.el.impl.parser.ELParserTreeConstants: **JJTEQUAL** - Static variable in interface org.jdesktop.el.impl.parser.ELParserTreeConstants: **JJTFALSE** - Static variable in interface org.jdesktop.el.impl.parser.ELParserTreeConstants: **JJTFLOATINGPOINT** - Static variable in interface org.jdesktop.el.impl.parser.ELParserTreeConstants: **JJTFUNCTION** - Static variable in interface org.jdesktop.el.impl.parser.ELParserTreeConstants: **jjtGetChild(int)** - Method in interface org.jdesktop.el.impl.parser.Node: This method returns a child node. **jjtGetChild(int)** - Method in class org.jdesktop.el.impl.parser.SimpleNode: **jjtGetNumChildren()** - Method in interface org.jdesktop.el.impl.parser.Node: Return the number of children the node has. **jjtGetNumChildren()** - Method in class org.jdesktop.el.impl.parser.SimpleNode: **jjtGetParent()** - Method in interface org.jdesktop.el.impl.parser.Node: **jjtGetParent()** - Method in class org.jdesktop.el.impl.parser.SimpleNode: **JJTGREATERTHAN** - Static variable in interface org.jdesktop.el.impl.parser.ELParserTreeConstants: **JJTGREATERTHANEQUAL** - Static variable in interface org.jdesktop.el.impl.parser.ELParserTreeConstants: **JJTIDENTIFIER** - Static variable in interface org.jdesktop.el.impl.parser.ELParserTreeConstants: **JJTINTEGER** - Static variable in interface org.jdesktop.el.impl.parser.ELParserTreeConstants: **JJTLESSTHAN** - Static variable in interface org.jdesktop.el.impl.parser.ELParserTreeConstants: **JJTLESSTHANEQUAL** - Static variable in interface org.jdesktop.el.impl.parser.ELParserTreeConstants: **JJTLITERALEXPRESSION** - Static variable in interface org.jdesktop.el.impl.parser.ELParserTreeConstants: **JJTMINUS** - Static variable in interface org.jdesktop.el.impl.parser.ELParserTreeConstants: **JJTMOD** - Static variable in interface org.jdesktop.el.impl.parser.ELParserTreeConstants: **JJTMULT** - Static variable in interface org.jdesktop.el.impl.parser.ELParserTreeConstants: **JJTNEGATIVE** - Static variable in interface org.jdesktop.el.impl.parser.ELParserTreeConstants: **jjtNodeName** - Static variable in interface org.jdesktop.el.impl.parser.ELParserTreeConstants: **JJTNOT** - Static variable in interface org.jdesktop.el.impl.parser.ELParserTreeConstants: **JJTNOTEQUAL** - Static variable in interface org.jdesktop.el.impl.parser.ELParserTreeConstants: **JJTNULL** - Static variable in interface org.jdesktop.el.impl.parser.ELParserTreeConstants: **jjtOpen()** - Method in interface org.jdesktop.el.impl.parser.Node: This method is called after the node has been made the current node. **jjtOpen()** - Method in class org.jdesktop.el.impl.parser.SimpleNode: **JJTOR** - Static variable in interface org.jdesktop.el.impl.parser.ELParserTreeConstants: **JJTPLUS** - Static variable in interface org.jdesktop.el.impl.parser.ELParserTreeConstants: **jjtree** - Variable in class org.jdesktop.el.impl.parser.ELParser: **jjtSetParent(Node)** - Method in interface org.jdesktop.el.impl.parser.Node: This pair of methods are used to inform the node of its parent. **jjtSetParent(Node)** - Method in class org.jdesktop.el.impl.parser.SimpleNode: **JJTSTRING** - Static variable in interface org.jdesktop.el.impl.parser.ELParserTreeConstants: **JJTTRUE** - Static variable in interface org.jdesktop.el.impl.parser.ELParserTreeConstants: **JJTVALUE** - Static variable in interface org.jdesktop.el.impl.parser.ELParserTreeConstants: **JJTVOID** - Static variable in interface org.jdesktop.el.impl.parser.ELParserTreeConstants: **JListAdapterProvider** - Class in org.jdesktop.swingbinding.adapters: **JListAdapterProvider()** - Constructor for class org.jdesktop.swingbinding.adapters.JListAdapterProvider: **JListAdapterProvider.Adapter** - Class in org.jdesktop.swingbinding.adapters: **JListBinding**<E,SS,TS> - Class in org.jdesktop.swingbinding: Binds a `List` of objects to act as the elements of a `JList`. **JListBinding(AutoBinding.UpdateStrategy, SS, Property<SS, List<E>>, TS, Property<TS, ? extends JList>, String)** - Constructor for class org.jdesktop.swingbinding.JListBinding: Constructs an instance of `JListBinding`. **JListBinding.DetailBinding** - Class in org.jdesktop.swingbinding: `DetailBinding` represents a binding between a property of the elements in the `JListBinding's` source `List`, and the values shown in the `JList`. **JSliderAdapterProvider** - Class in org.jdesktop.swingbinding.adapters: **JSliderAdapterProvider()** - Constructor for class org.jdesktop.swingbinding.adapters.JSliderAdapterProvider: **JSliderAdapterProvider.Adapter** - Class in org.jdesktop.swingbinding.adapters: **JSpinnerAdapterProvider** - Class in org.jdesktop.swingbinding.adapters: **JSpinnerAdapterProvider()** - Constructor for class org.jdesktop.swingbinding.adapters.JSpinnerAdapterProvider: **JSpinnerAdapterProvider.Adapter** - Class in org.jdesktop.swingbinding.adapters: **JTableAdapterProvider** - Class in org.jdesktop.swingbinding.adapters: **JTableAdapterProvider()** - Constructor for class org.jdesktop.swingbinding.adapters.JTableAdapterProvider: **JTableAdapterProvider.Adapter** - Class in org.jdesktop.swingbinding.adapters: **JTableBinding**<E,SS,TS> - Class in org.jdesktop.swingbinding: Binds a `List` of objects to act as the rows of a `JTable`. **JTableBinding(AutoBinding.UpdateStrategy, SS, Property<SS, List<E>>, TS, Property<TS, ? extends JTable>, String)** - Constructor for class org.jdesktop.swingbinding.JTableBinding: Constructs an instance of `JTableBinding`. **JTableBinding.ColumnBinding** - Class in org.jdesktop.swingbinding: `ColumnBinding` represents a binding between a property of the elements in the `JTableBinding's` source `List`, and a column in the table. **JTextComponentAdapterProvider** - Class in org.jdesktop.swingbinding.adapters: **JTextComponentAdapterProvider()** - Constructor for class org.jdesktop.swingbinding.adapters.JTextComponentAdapterProvider: **JTextComponentAdapterProvider.Adapter** - Class in org.jdesktop.swingbinding.adapters

---


|  |  |  |
| --- | --- | --- |
| **Overview** \| Package \| Class \| Use \| **Tree** \| **Deprecated** \| **Index** \| **Help** | |  |
| **PREV LETTER**   **NEXT LETTER** | **FRAMES**    **NO FRAMES**     **All Classes** |  |


A B C D E F G H I J K L M N O P Q R S T U V W 

---
